# Focal Myositis Localised in Gastrocnemius Muscle: is it Different from Isolated Gastrocnemius Myositis? A Case Report

**DOI:** 10.5704/MOJ.2111.022

**Published:** 2021-11

**Authors:** IS Son, JS Kim, SJ Yoo, MS Kang, CL Hyun

**Affiliations:** 1 Department of Orthopedic Surgery, Jeju National University College of Medicine and Graduate School of Medicine, Jeju, South Korea; 2 Department of Rhematology, Jeju National University College of Medicine and Graduate School of Medicine, Jeju, South Korea; 3 Department of Orthopedic Surgery, Bumin Hospital Seoul, Seoul, South Korea; 4 Department of Pathology, Jeju National University College of Medicine and Graduate School of Medicine, Jeju, South Korea

**Keywords:** focal myositis, isolated gastrocnemius myositis, pseudotumour

## Abstract

Focal myositis is a rare disease defined by an isolated inflammatory pseudotumour usually restricted to one skeletal muscle. Approximately, 250 cases of focal myositis have been described in the literature, and two recent large cohorts have been used to help in the diagnosis. Isolated gastrocnemius myositis, a rare immune-mediated condition, is a diagnostic entity used by internal medicine clinician in the gastrocnemius myalgia syndrome associated with Crohn’s disease (CD). However, focal myositis and isolated gastrocnemius myositis with Crohn's disease share clinical, haematological, pathological, and radiological similarities. We present a case of unilateral focal myositis of the gastrocnemius muscle in a patient with no underlying diseases, including Crohn’s disease. At clinical evaluation, we encountered a challenge in differentiating between focal myositis and the isolated gastrocnemius myositis of Crohn’s due to similarities in clinical manifestation. We attempt to clarify focal myositis and isolated gastrocnemius myositis through our case report and a review of literature.

## Introduction

Focal myositis (FM) is characterised by a monofocal mass usually restricted to one skeletal muscle compartment, and was first described in 1977. There have been approximately 250 cases in the literature worldwide^1,2^. Previous literature on FM indicated its possible causes associated with radiculopathy, neoplasm, trauma, infection, and autoimmune diseases, most commonly involving skeletal muscles of lower extremity in adductor muscle, vastus lateralis, and gastrocnemius^3^. Another disease entity associated with focally occurring myositis in a single muscle compartment is the isolated gastrocnemius myositis (IGM), an extremely rare form of extra-intestinal manifestation associated with Crohn’s disease (CD)^4,5^. FM and IGM share similar clinical, radiological, and histological features and a benign self-limited course of disease.

We describe a case of unilateral FM occurring in the gastrocnemius muscle in a 48-year-old female with no other underlying disease, together with a review of the literature. A written informed consent for participation and publication was obtained from the patient in compliance with the Declaration of Helsinki.

## Case Report

**Fig. 1:**
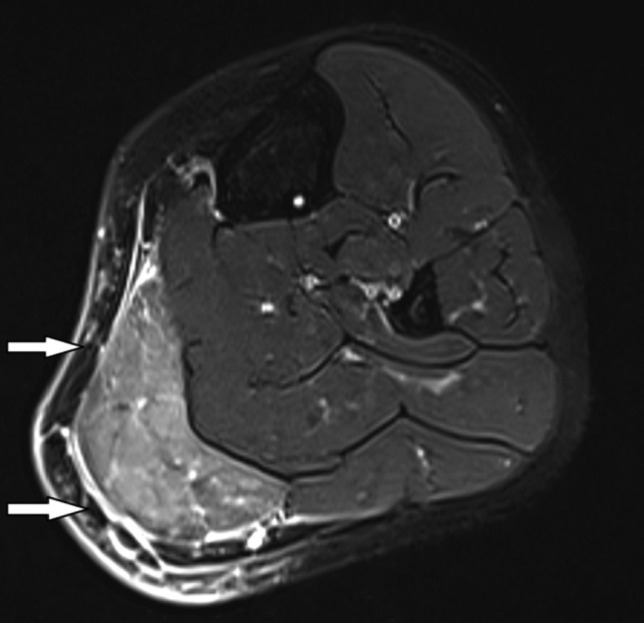
Left leg T2 weighted MRI coronal image showed increased signal intensity on T2 weighted image and diffuse enhancement in gastrocnemius muscle (white arrow).

**Fig. 2:**
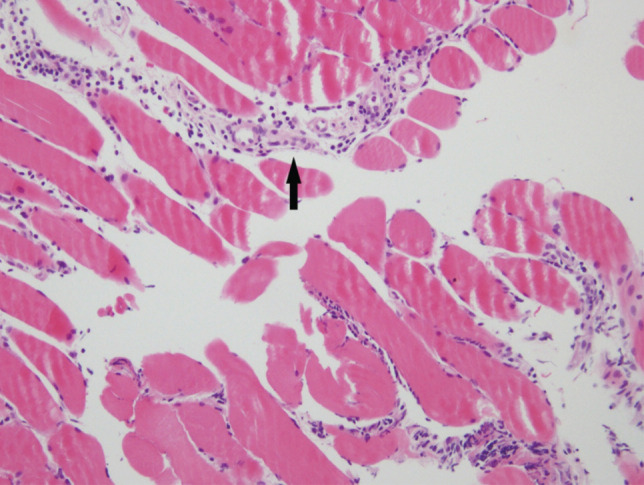
Microscopic findings in x200. Microscopically, the fibers of gastrocnemius muscle revealed mild size variation and inflammatory cell infiltrations in the endomysium and perivascular area (black arrow). Degeneration and regeneration of muscle fibers associated with interstitial inflammation were also seen. Internal nuclei and features of vasculitis were not prominent.

A 49-year-old female patient with no underlying diseases visited the outpatient clinic with a chief complaint of pain around the left calf without a history of trauma, for the duration of one week. The patient exhibited a burning sensation localised within the left calf, and discomfort on ambulation. Physical examination revealed prominent warmth and acute tenderness on the medial aspect of the left gastrocnemius, without fever or neuromuscular deficits. The laboratory examination indicated mildly elevated erythrocyte sedimentation rate (ESR) of 58mm/hr and C-reactive protein (CRP) of 0.81mg/dL without leucocytosis. Initially upon clinical diagnosis of cellulitis, intravenous (IV) cefazolin (2 grams q 8 hours) was administered after blood culture was performed, but no symptom resolution was evident after two days. Computed tomography (CT) angiography and revealed no definite stenosis or occlusion indicative of thrombus or embolism in the blood vessels. Magnetic resonance imaging (MRI) showed high signal intensity in the medial head and distal portion of the lateral head in the left gastrocnemius muscle with subcutaneous oedema on T2-weighted images (Fig. 1). After a transfer to the department of rheumatology, additional blood work up, including creatinine kinase (CK), ferritin, and leucocyte dehydrogenase (LDH), showed no abnormal findings, and antibody screening tests, includcing antinuclear antibodies (ANA) titration, anti-neutrophil cytoplasmic antibody (ANCA), were negative. On a clinical assumption of IGM, a muscle biopsy was performed that indicated mild lymphocytic infiltration without evidence of vasculitis (Fig. 2). In the search for the possible manifestation of CD, gastroscopy and colonoscopy showed no evidence of inflammatory bowel diseases. With a clinical diagnosis of FM, the patient recovered fully with oral prednisolone therapy and was followed-up for one year without recurrence.

## Discussion

FM is a rare soft tissue pseudotumour localised in a single musculoskeletal compartment, most commonly involving in adductor muscle and lateralis vastus in lower legs without specific systemic manifestations. It is associated with immune-mediated causes including autoimmune disease and neoplasm; non-immune mediated causes such as infection, trauma, and radiculopathy; and iatrogenic and idiopathic causes^2,5^. The nature of the clinical course is benign, self-limiting, and responsive to immune-modulating agents^2,3^. Haematological assessments are mostly unremarkable, and focalised inflammatory abnormalities in MRI with myogenic or neurogenic patterns in electromyography (EMG) may help its diagnosis but are often nonspecific. In addition, pathological investigation reveals myopathic changes and inflammatory infiltrates, and immunohistologic staining (IHS) may help identify more detailed pathological patterns^1^.

In a cohort study of 115 cases with FM, there were 22 solitary FM (19.1%) in the gastrocnemius; and in another study, there were 26 cases (70%) with mono-focal FM in the lower limbs^1,2^. Among many autoimmune causes in FM such as Behcet disease, SLE, and systemic sclerosis, there have been no reports associated with CD^2,3^. IGM, previously reported only in six case reports, is an extremely rare extra-intestinal manifestation in CD; however, its clinical manifestation shows nonspecific findings in laboratory, hematological, radiological, histological, and EMG assessment, as in FM^4,5^. In addition, IHS revealed findings associated with CD68-, CD4-, and CD8-positive infiltrates, in necrotic and regenerating muscle fibres^2^. Under these circumstances, the authors questioned whether IGM is equivalent to FM occurring solitarily in gastrocnemius associated with Crohn’s disease.

Despite the first impression of cellulitis, haematological assessment and a lack of response to empirical antibiotics led us to investigate for other possible diagnoses. The radiological and pathological assessment confirmed FM localised solely in the gastrocnemius muscle, and gastroscopy and colonoscopy performed in search for its potential association with CD showed no evidence of inflammatory bowel diseases. In clinical evolution, the authors believed that IGM with CD is also another type of FM. Previous literature on aetiology of FM described various autoimmune causes. However, IGM was exclusively confined to its diagnostic connection with CD despite similar clinical characteristics with FM^2^. Although many treatment options including glucocorticoids, colchicine, methotrexate, and immunosuppressants such as azathioprine and intravenous immunoglobulins have been reported, no consensus has been reached on the gold standard of treatment. Single or combined glucocorticoids with immunosuppressants was also evident in the current study^2,5^. In myositis solitarily occurring in gastrocnemius, endoscopy and biopsy are helpful to rule out rheumatic diseases and inflammatory bowel diseases due to the possibility of temporal discrepancy in manifestation of extra-intestinal and intestinal symptoms in CD^4^.

The limitation of the study is that we were not able to compare the case of FM to IGM; therefore, we conducted a comparison between FM and IGM via a review of the literature. However, in the course of clinical evolution, we proposed that IGM with CD may also be a type of FM, sharing equivalent clinical, radiological, and histological characteristics.

Because FM is associated with various aetiologies, gastrointestinal endoscopy is a way to look for an unrecognised cause of focalised pseudotumour occurring in the single musculoskeletal compartment of the lower extremity. However, due to their rare occurrences of FM and IGM, further research with a long-term follow-up is required to compare clinical features and progression.
